# P03-012-B - Lupus erythematous chronicus: a new etiology of macrocheilitis

**DOI:** 10.1186/1546-0096-11-S1-A209

**Published:** 2013-11-08

**Authors:** M Mael-Ainin, H Boudhir, A Saidi, B Hassam, K Senouci

**Affiliations:** 1 Dermatologie - Vénérologie, Centre Hospitalier Universitaire ibn Sina, Rabat, Morocco; 2 Centre of Anatomical Pathology, United Nations, Rabat, Morocco

## Introduction

Macrocheilitis etiologies are diverse, particularly granulomatous diseases. We report the first case occurring in the context of lupus erythematous chronicus.

## Objectives

To show the importance of investigating lupus in etiological diagnosis of macrocheilitis.

## Methods

Mrs. E.F a 30 years old woman, was followed up, since 2003, for a lupus erythematous chronicus. In 2010, she presented a painful cheilitis of the upper lip that gradually increases in volume. Clinical examination showed a scaly macrocheilitis, the rest of the clinical examination was within normal limits. The labial biopsy showed a dyskeratotic hyperkeratosis, keratotic plugs, a pseudo-epitheliomatous hyperplasia, an inflammatory infiltrate of the chorion without granuloma, a direct immunofluorescence that was negative, antinuclear antibodies were positive at 1/80, anti-native DNA antibodies were negative. The systematization work up was unremarkable. The diagnosis of lupus erythematous chronicus was retained. The patient was put under Chloroquine at 4mg/kg/day dose causing total regression of symptoms within 6 months without recurrence for one year.

## Results

Macrocheilitis etiologies are diverse, mainly of granulomatous nature especially Melkersson Rosenthal syndrome, Crohn's disease or sarcoidosis. Macrocheilitis in the lupus disease has never been reported in the literature. Indeed, lupus erythematous chronicus manifest in keratotic whitish lesions of the vermilion, systemic lupus in erosive and crusting cheilitis. These lesions can be the initial symptom or occur during the evolution of the disease.

The etiologic diagnosis of macrocheilitis can rely on several clinical and paraclinical data. In our case, the antecedent of the lupus disease of the patient, the clinical exam, the histology of lip biopsy and the antinuclear antibodies positivity have retain lupus erythematosus as an etiology of this cheilitis.

The treatment of macrocheilitis is difficult; various therapies have been used with varying results, including oral or intralesional corticosteroids, antimalarials, immunosuppressives, antibiotics and biotherapies, or in some cases cheiloplasty reduction. In our case, improvement was obtained under Chloroquine at a dose of 4mg/kg/day.

## Conclusion

To our knowledge, we present the first case of cheilitis occurring in the context of erythematosus lupus, this exceptional etiology is worth being known as it allows for an appropriate treatment.

## Competing interests

None Declared.

